# Plasma *p*‐tau217 Performance for Detection of Alzheimer's Disease Neuropathology in Underrepresented Populations

**DOI:** 10.1002/alz70856_105551

**Published:** 2026-01-07

**Authors:** Yoav D Piura, Christian Lachner, Alicia Algeciras‐Schimnich, Daniel Figdore, Joshua A Bornhorst, Paula Aduen, Leah Schecter, Neill R. Graff‐Radford, Gregory S Day

**Affiliations:** ^1^ Mayo Clinic in Florida, Jacksonville, FL, USA; ^2^ Mayo Clinic, Rochester, MN, USA

## Abstract

**Background:**

Black and Latino Americans experience a disparate dementia burden yet remain underrepresented in dementia research, including clinical trials designed to slow or prevent symptomatic Alzheimer's disease (AD). Accessible and non‐invasive AD blood biomarkers may improve identification and recruitment of underrepresented participants into AD clinical trials. There is a clear need to assess the performance of emergent plasma AD biomarkers for the detection of AD neuropathology in cognitively normal Black and Latino Americans.

**Method:**

Cognitively normal Black and Latino participants underwent cognitive evaluations and PET (amyloid‐PiB, tau‐flortaucipir) imaging at Mayo Clinic in Florida. Plasma *p*‐tau217 was measured using Fujirebio Lumipulse assays. We evaluated the diagnostic performance of a *p*‐tau217 threshold (≥0.186 pg/mL) known to identify non‐Hispanic White participants with cognitive impairment and “positive” amyloid PET scans (>25 Centiloids). Next, we derived an optimized threshold to identify participants with “positive” amyloid PET using Youden's method.

**Result:**

The cohort included 58 Black (55%) and 47 Latino‐White (45%) well‐educated (mean 16.1±2.3 years) individuals, with mean age 64.7±8.8 years, and slight female bias (63%). Plasma *p*‐tau217 concentrations increased with age (rho=0.269, *p* = 0.004) and declined with MoCA scores (rho=‐0.225, *p* = 0.027). Sixty‐nine participants completed amyloid‐ and tau‐PET imaging. Plasma *p*‐tau217 concentrations increased with amyloid‐ (rho=0.254, *p* = 0.044) but not with tau‐PET standardized uptake value ratio (rho=0.127; *p* = 0.334). *p*‐tau217 concentrations were ≥0.186 pg/mL in 10/69 participants (14%), including 3/3 amyloid‐ and tau‐PET positive (A+T+) participants, 3/8 A+T‐ participants (sensitivity: 55%), and 4/58 A‐T‐ participants (specificity 93%). An optimized *p*‐tau217 threshold of ≥0.118 pg/mL identified 10/11 A+T± participants (sensitivity 91%), and 16/58 A‐T‐ participants (specificity 73%). In A‐T‐ participants, estimated glomerular filtration rate was lower in participants with ‘false positive’ elevations in plasma *p*‐tau217 (mean difference: 67 vs 81 ml/min/1.73m^2^, *p* = 0.004), emphasizing the need for caution when interpreting plasma *p*‐tau217 concentrations in patients with decreased kidney function.

**Conclusion:**

Plasma *p*‐tau217 thresholds validated in participants with symptomatic AD (≥0.186 pg/mL) failed to identify early AD neuropathology in 5/8 cognitively normal Black and Latino participants. Optimized thresholds are required to improve detection of preclinical AD in Black/Latino individuals.

